# The effects of an 8-week computerized cognitive training program in older adults: a study protocol for a randomized controlled trial

**DOI:** 10.1186/s12877-018-0730-6

**Published:** 2018-01-30

**Authors:** Lisanne F. ten Brinke, John R. Best, Rachel A. Crockett, Teresa Liu-Ambrose

**Affiliations:** 1 Aging, Mobility, and Cognitive Neuroscience Laboratory, Department of Physical Therapy, Djavad Mowafaghian Centre for Brain Health, University of British Columbia, 2215 Wesbrook Mall, Vancouver, BC V6T 1Z3 Canada; 2 Aging, Mobility, and Cognitive Neuroscience Laboratory, Department of Physical Therapy, Djavad Mowafaghian Centre for Brain Health, Centre for Hip Health and Mobility, University of British Columbia, 2215 Wesbrook Mall, Vancouver, BC V6T 1Z3 Canada

**Keywords:** Computerized cognitive training, Community-dwelling older adults, Mild cognitive impairment, Cognitive function, Magnetic resonance imaging

## Abstract

**Background:**

Given the world’s aging population, it is important to identify strategies that promote healthy cognitive aging and minimize cognitive decline. Currently, no curative pharmaceutical therapy exists for cognitive impairment and dementia. As a result, there is much interest in lifestyle approaches. Specifically, complex mental activity, such as cognitive training, may be a promising method to combat cognitive decline in older adults. As such, the industry of commercial computerized cognitive training (CCT) applications has rapidly grown in the last decade. However, the efficacy of these commercial products is largely not established. Moreover, exercise is a recognized strategy for promoting cognitive outcomes in older adults and may augment the efficacy of computerized cognitive training applications. Therefore, we propose a proof-of-concept randomized controlled trial (RCT) to examine the effect of a commercial CCT program in community-dwelling older adults.

**Methods:**

An 8-week RCT to examine the effect of a commercial CCT program, alone and preceded by a 15-min brisk walk, on cognitive function and explore the underlying neural mechanisms in adults aged 65–85 years old. Participants will be randomized to one of three intervention groups: 1) Computerized cognitive training (FBT); 2) A 15-min brisk walk followed by computerized cognitive training (Ex-FBT); or 3) A combination of educational classes, sham cognitive training, and balanced and tone exercises (active control, BAT). Participants in all intervention groups will attend three one-hour classes per week over the course of the intervention. Participants will be assessed at baseline, trial completion, and 1-year post study completion (1-year follow-up).

**Discussion:**

If results from this study show benefits for cognition at trial completion, CCT programs, alone or in combination with walking, might be a strategy to promote healthy cognitive aging in older adults. In addition, results from the 1-year follow-up measurement could provide important information regarding the long-term benefits of these CCT programs.

**Trial registration:**

ClinicalTrials.gov Protocol Registration System: NCT02564809; registered September 1, 2015.

## Background

The world’s population is aging, and the promotion of active aging is a global priority [1]. Cognitive impairment and dementia are now the leading cause of disablement and death in later life. The incidence of dementia is rising rapidly, and over 47 million people worldwide are diagnosed with dementia and this number is expected to triple by 2050 [1]. As an effective treatment or cure for dementia remains elusive, there are increased efforts to establish the efficacy of non-pharmaceutical strategies, such as targeted exercise training and cognitive training, on cognitive health in older adults. Even when an effective pharmacological therapy is available, lifestyle approaches (i.e., exercise, nutrition, and cognitive training) can be used as a complementary approach, as lifestyle interventions result in multidimensional benefits [2].

Interest in strategies such as cognitive training, a form of complex mental activities, has increased over the last decade. Tasks aimed to train for example executive functions, memory, or learning a language are considered complex mental activities, as long as they challenge an individual cognitively [3]. Improvements in cognitive function, such as episodic memory (e.g., delayed recall), were found in older adults who participated in videogames [4] or computer lessons [5]. Moreover, auditory perception training for 6 weeks, 1 h per day, resulted in improvements in problem solving and reasoning [6], which is considered to be positive far transfer. Thus, besides improvements in the trained domains, cognitive training could also show benefits of transfer [4, 6]. Aside from immediate benefits, the ACTIVE study [7] found that ten years post intervention, participants who received either speed-of-processing training or reasoning training for 5–6 weeks maintained effects of targeted cognitive abilities (i.e., speed-of-processing, reasoning). A meta-analysis of human cohort studies demonstrates that the amount of time involved in complex mental activities in early, mid- and late-life, was associated with a reduction in dementia incidence in later life [8]. Specifically, they found that increased complex mental activity in later life was associated with lower dementia rates, independent of other predictors, where more involvement in complex mental activities was found to lower dementia risk [8].

One example of complex mental activity that received increasing attention as a strategy to promote healthy cognitive aging is computerized cognitive training (CCT). The number of commercialized CCT programs has increased rapidly over the last years. A meta-analysis of CCT in older adults showed that CCT is able to improve overall cognitive function, memory (verbal, non-verbal), processing speed, working memory and visuospatial skills [9]. No improvements were found for executive functions and attention [9]. A recent randomized controlled trial (RCT) comparing multidomain CCT with an active control group found improvements in global cognition, memory and processing speed [10]. Improvements in memory and processing speed were maintained at 1-year follow up, indicating maintenance of CCT benefits [10]. Thus, CCT is a promising strategy to promote healthy cognitive aging, and is also a feasible strategy for those who are limited in their abilities to participate in other lifestyle strategies, such as exercise.

Aerobic exercise is a promising strategy to promote cognitive health, while benefiting cardiovascular function at the same time [11]. Research shows that aerobic exercise, such as walking, could benefit cognitive function such as executive functions (e.g., inhibition, processing speed), memory [11–14], as well as brain structure [13, 15] and function [16]. As both exercise and cognitive training are promising strategies to prevent or delay cognitive decline [17], perhaps by combining them the benefit may be increased. Importantly, whereas aerobic exercise can facilitate neuroplasticity by increasing the number of newly formed neurons, additional experience-dependent cognitive activity is necessary to promote synaptic plasticity and the survival and functional integration of the newly formed neurons into neural networks [18–21]. Moreover, due to the transient nature of the upregulation of neurotrophic factors [22] it has been suggested that cognitive training preferably takes place in temporal proximity to exercise training [23].

The objective of the current proof-of-concept RCT will be to examine the effect of CCT, alone and preceded by a 15-min brisk walk, on cognitive function and to explore the underlying neural mechanism in community dwelling older adults. Therefore, our aim is four-fold: 1) To compare the effects of an 8-week CCT program (i.e., Fit Brains***®*** Training: FBT), as well as the effects of a 15-min brisk walk prior to FBT (i.e., Ex-FBT), with an active control (i.e., Balanced And Toned, BAT) on cognitive performance in older adults aged 65–85 years old; 2) Using structural and functional Magnetic Resonance Imaging (MRI), to explore the effect of FBT and Ex-FBT compared with BAT on brain structure and function; 3) To explore whether the effects of FBT and Ex-FBT are moderated by baseline cognitive status (i.e., Mild Cognitive Impairment (MCI) versus non-MCI); 4) To explore whether Ex-FBT has additional benefits compared with FBT; and 5) To explore whether potential benefits from CCT are maintained at 1-year follow-up.

## Methods

### Trial design

**Fig. 1:**
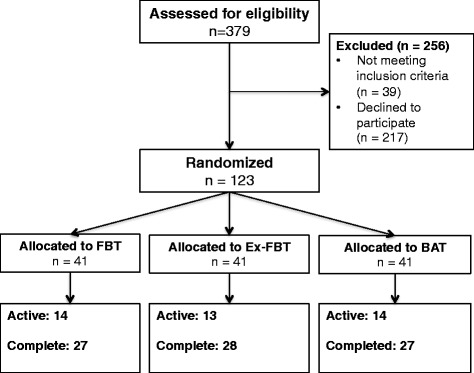
Overview of participant flow

vThis proof-of-concept RCT in community-dwelling older adults will have three experimental arms. We will include 120 community-dwelling adults aged 65–85 years old who will be randomized to one of three experimental groups: 1) Computerized cognitive training (FBT); 2) Exercise plus computerized cognitive training (Ex-FBT); or 3) Balanced and Toned (BAT, i.e., active control, see Fig. 1). There will be three measurement sessions: baseline, trial completion (i.e., 8 weeks), and 1-year follow-up. The study protocol follows the Consolidated Standard of Reporting Trials (CONSORT) statement [24] and basic requirements from the Standard Protocol Items: Recommendations for Interventional Trials (SPIRIT) [25]. The trial is registered at ClinicalTrials.gov (NCT02564809).

### Study setting

The study will be conducted at two locations in Metro Vancouver, BC (Canada): the Djavad Mowafaghian Centre for Brain Health at the University of British Columbia (UBC) and the Centre for Hip Health and Mobility at Vancouver General Hospital (VGH).

### Participants

Participants will be recruited from the community (Greater Vancouver, BC Canada) as well as through our database of previous research participants. Individuals showing interest in the study via advertisements in community centres or local newspapers will receive a short summary of the study and if still interested, will be screened over the telephone to determine eligibility. Participants from previous studies in our laboratory who expressed interest in future studies will be contacted either via mail or email.

### Eligibility

#### Inclusion criteria

For this study, we will include individuals who: 1) are aged between 65 and 85 years; 2) completed high school education; 3) live in their own home; 4) read, write, and speak English with acceptable visual and auditory acuity; 5) have preserved general cognitive function as indicated by a Mini-Mental State Examination [26] score of ≥24/30; 6) score > 6/8 on the Lawton and Brody Instrumental Activities of Daily Living Scale [27]; 7) are not expected to start or are stable on a fixed dose of anti-dementia medications (e.g., donepezil, galantamine, etc.) during the 8-week study period; 8) are able to walk independently; 9) are suitable to engage in 15 min of brisk walking based on the Physical Activity Readiness Questionnaire [28]; and 10) provide a personally signed and dated informed consent document indicating that the individual (or a legally acceptable representative) has been informed of all pertinent aspects of the study.

#### Exclusion criteria

We will exclude individuals who: 1) are diagnosed with dementia of any type; 2) are clinically suspected to have a neurodegenerative disease as the cause of MCI that is not AD, vascular dementia (VaD), or both (e.g. multiple sclerosis, Parkinson’s disease, Huntington’s disease, fronto-temporal dementia, etc.); 3) have clinically significant peripheral neuropathy or severe musculoskeletal or joint disease that impairs mobility, as determined by his/her family physician; 4) are taking medications that may negatively affect cognitive function, such as anticholinergics, including agents with pronounced anticholinergic properties (e.g., amitriptyline), major tranquilizers (i.e., typical and atypical antipsychotics), and anticonvulsants (e.g., gabapentin, valproic acid, etc.); and 5) are planning to participate, or already enrolled in, a concurrent clinical drug trial.

A subset of participants will undergo MRI scanning. For this MRI subset, we will exclude individuals who do not meet the specific scanning requirements of the UBC MRI Research Centre. Specifically, we will exclude anyone with: pacemaker, brain aneurysm clip, cochlear implant, surgery or tattoos within the past 6 weeks, electrical stimulator for nerves or bones, implanted infusion pump, history of any eye injury involving metal fragments, artificial heart valve, orthopedic hardware, other metallic prostheses, coil, catheter or filter in any blood vessel, ear or eye implant, bullets, or other metallic fragments.

### Classification by baseline cognitive status

To explore whether the intervention effects (i.e., FBT and Ex-FBT) are moderated by baseline cognitive status, we will classify individuals based on their baseline Montreal Cognitive Assessment (MoCA) [29]. The MoCA is a 30-point test that covers multiple cognitive domains [29]. The MoCA has been found to have good internal consistency and test-retest reliability and was able to correctly identify 90% of a large sample of individuals with MCI from two different clinics [29]. Thus, participants with a baseline MoCA score ≤ 26/30 will be classified as probable MCI and those with a MoCA score > 26/30 will be classified as cognitively normal.

### Interventions

For the 8-week intervention period, all participants will be asked to come to the study location (i.e., VGH or UBC) 3 times per week for 1 h. Thus, all participants will attend 24 1-hour classes at VGH or UBC. These classes will have a set time, and thus after randomization participants will come in on Monday, Wednesday and Friday at the same time each day. Over the course of the four study cohorts, group times will be kept consistent (+/− 15 min). In addition, study staff will be kept consistent over all four cohorts to ensure training consistency. Depending on group size, students/staff will help facilitate study classes to meet the participants’ needs.

#### Fit brains® training (FBT)

Participants randomized to the FBT group will be required to attend 3 formal training sessions per week, for 8 weeks, at the Djavad Mowafaghian Centre for Brain Health (UBC) or the Centre for Hip Health and Mobility (VGH). Each session will be for 60 min. Additionally, participants will be asked to complete 3 1-hour training sessions at home per week. Thus, FBT participants will complete a total of 48 h of cognitive training over the 8-week intervention.

There is currently no consensus as to the “best dosage”. However, we based our proposed dosage on the collective work by Strenziok and colleagues [6], Basak and colleagues [4], Engvig and colleagues [30], and Smith and colleagues [31]. Overall, the total number of training hours ranged from 23.5 h to 40 h, each training session ranged from 60 min to 90 min, and total intervention period ranged from 5 weeks to 8 weeks. Importantly, the study population included by Envig and colleagues [30] (i.e., older adults with subjective memory complaints) is the most similar to our target population. They employed an 8-week intervention period with one formal training session of 90 min and five home-based sessions. Each home-based session was approximately 30 min. Thus, their total number of training hours was ~ 32 h (12 h of formal training and 20 h of at home training). Notably, Envig and colleagues [30] demonstrated that after 8-weeks of training, there was significant improvement in verbal memory (i.e., long verbal delay recall) and increases in gray matter volumes. To be conservative, we increased our total number of training hours to 48 as data extracted from existing Fit Brains***®*** subscribers suggest that compared with young adults, older adults may require more frequent cognitive training to maintain benefit [32].

Fit Brains***®***, a program by Rosetta Stone Inc., offers 59 different training games, of which 38 are available on a mobile platform (e.g., iPad). The games are designed to be targeting one of six cognitive domains – focus, speed, memory, visual, problem solving, and language. The majority of the games last exactly 60 s during which individual aims to answer as many questions as quickly and accurately as possible. Other games have a set number of trials the participants have to complete before moving on to the next game. The difficulty of the game increases after each correct answer. Each game has three levels of difficulty: 1) novice; 2) intermediate; and 3) advanced.

During the FBT intervention, all participants will begin the training at the beginner level. Difficulty will increase throughout the intervention period based on their performance. At the end of each training session, FBT game progress will be saved and participants will begin the next session at that point. Each block of games will consist of 5 games. The first 5 blocks of games will be prescribed to offer an introduction to the user. After that, the sequencing of the games will be random, where each block will consist of games that need the most attention (i.e., games that showed the lowest performance), and games that will be randomly selected based on a set algorithm. Game performance will be recorded for each participant. Moreover, for their training sessions at home, participants will be asked to train at the same time of the day as their classes at VGH/UBC.

#### Exercise + fit brains® training (ex-FBT)

Participants randomized to the Ex-FBT group will be required to attend 3 formal training sessions per week, for 8 weeks, at the Djavad Mowafaghian Centre for Brain Health (UBC) or the Centre for Hip Health and Mobility (VGH). Each session will be for 1 h. Participants will start the training with a 15-min walk outside. Participants will monitor the intensity of their walk using the 20-point Borg’s Rating of Perceived Exertion [33]. For the first two weeks the participants will aim for a 10–11 on the Borg scale (i.e., between very light and fairly light). The aim for weeks 3 and 4 will be to reach for 12–13 on the Borg scale (i.e., up to somewhat hard). During the remaining 4 weeks the participants will aim for 13–14 on the Borg scale (i.e., somewhat hard). The 15-min walk will be followed by a 45-min Fit Brains***®*** training session (see FBT program, mentioned above) on the iPad. Additionally, participants will be asked to complete 3 1-h training sessions at home (i.e., 15-min walk followed by 45-min of FBT). The participants will be recording their Borg-scale scores and the number of steps they walked during their 15-min walks on a calendar that will be provided at the start of the study.

#### Balanced and toned (BAT)

Participants randomized to the BAT group will be required to attend 3 formal 1-h training sessions per week, for 8 weeks, at the Djavad Mowafaghian Centre for Brain Health (UBC) and/or the Centre for Hip Health and Mobility (VGH). Specifically, the BAT participants will complete a total of 8 h of sham cognitive training, 8 h of sham exercise training, and 8 h of education regarding brain health over the 8-week training.

We have largely designed the sham cognitive training of the BAT protocol based on the work of Baniqued and colleagues [34] who examined the nature of cognitive abilities tapped by casual online games. They identified online games that largely tapped solely into visuo-motor speed, such as Alphattack and Crashdown. Alphattack requires players to prevent bombs from landing by pressing the character specified by the approaching bomb (source: miniclip.com). Crashdown requires players to prevent the wall from reaching the top of the display by clipping on three or more adjacent same-coloured bricks to remove them (source: miniclip.com). As these online games do not significantly tap into memory abilities, we use similar online games in our BAT protocol. In addition to exercises on the iPad we include group-based games, such as drawing using both their dominant and non-dominant hand, writing captions on cartoons, and word games.

The exercise component of the BAT program will consist of once weekly balance and tone classes. The exercise program will be led by certified fitness instructors (i.e., CPR certified and NCAA certified or equivalent) and includes stretching exercises, range of motion exercises, basic core-strength exercises including kegals (i.e., exercises to strengthen the pelvic floor muscles), balance exercises and relaxation techniques. Key balance exercises include Tai Chi-based forms (i.e., Crane, Tree Pose), tandem stand, tandem walking, and single leg stance (eyes open and closed). Previous use of this protocol showed no improvements of cognitive functioning as a result of the BAT program [35]. These sessions will be held at the Centre for Hip Health and Mobility.

Additionally, once a week the participants will attend educational classes. For the first four 1-hour education sessions, participants will attend lectures relating to brain health, such as sleep and goal setting. During the remaining four weeks, participants will create their individual photo book using the iPad.

#### Adherence

Participants’ adherence to the interventions will be recorded using three methods. First, class attendance will be recorded by study team members. Second, monitoring CCT training at home will be done by the study team using the number of minutes trained per day registered by the program and provided by Rosetta Stone Inc. Third, we will ask participants to record their training minutes on a homework calendar provided by the study team.

### Outcome measures

All participants in the current study will attend three measurement sessions at VGH: baseline, trial completion, and 1-year follow-up. Each visit to VGH will be up to 3 h in duration. In addition, if interested and eligible, a subset of participants will attend two MRI scans (1.5 h per appointment) at UBC over the duration of the study (i.e., at baseline and trial completion). Our trained research staff, which will assess enrolled participants at baseline, trial completion, and 1-year follow-up, will be blinded to group allocation.

#### Descriptive measures

At baseline, general health, demographics, socioeconomic status, and education will be ascertained by a questionnaire. Descriptive measures such as age in years, standing and sitting height in centimetres, mass in kilograms, and waist and hip circumference in centimetres will be obtained.

##### Global cognitive function

Global cognitive function will be measured using both the Mini-Mental State Examination (MMSE) and the Montreal Cognitive Assessment (MoCA). The MoCA is a valid and reliable measure [29], and assesses eight cognitive domains such as attention, concentration, executive functions, memory, language and visuoconstructional skills. The total possible score is 30 points; a score of less than 26 points indicates MCI. The MoCA has with a score of 26 a 90% sensitivity to for detecting MCI [29].

##### General health, falls history, and socioeconomic status

We will administer questionnaires to obtain information about their level of education, employment status and general health information (e.g., medication, fall and fracture history).

##### Instrumental activities of daily living scale

The Lawton and Brody Instrumental Activities of Daily Living (IADL) [27] Questionnaire will be administered to assess the participants’ ability to perform tasks of daily living such as housekeeping, laundry, transportation, and management of finances. The questionnaire looks at eight different types of daily activities, and therefore it has a maximum achievable score of eight.

##### Co-morbidity

To assess the presence of any medical conditions, the functional comorbidity index (FCI) [36] will be used. In this scale, which contains 18 conditions, participants will indicate whether the condition is present currently, in the past or not at all.

##### Cognitive activity over lifetime

At baseline, we will administer a questionnaire focusing on lifetime stimulation of cognitively stimulating activities in a subset of participants [37]. This questionnaire measures the involvement in cognitively stimulating activities during their lifetime, namely at age 6, 12, 18, 40, and at their present age. Cognitively stimulating activities include visits to the library, read a newspaper, read a book, write a letter, and play a game. The involvement on all 25 items included will be rated on a 5-point scale, with 1) Once per year or less; 2) Several times per year; 3) Several times per month; 4) Several times per week; or 5) Every day or nearly every day.

#### Primary outcome: Episodic memory

Our primary cognitive outcome will be (verbal) episodic memory as measured by the Rey Auditory Verbal Learning Test (RAVLT) [38]. The RAVLT is a valid, reliable, and widely used instrument of (verbal) episodic memory. Notably, a 2013 prospective study showed that among a combination of neuropsychological, neuroimaging, and cerebrospinal fluid markers, RAVLT performance was the best individual predictor of MCI conversion to dementia [39]. For the RAVLT, a list of 15 common words (List A) will be read to participants five times. Immediately after each time, they will be asked to recall as many words as possible. After the fifth trial, an interference list (List B) will be presented, after which participants will be asked to spontaneously recall the words form the original list (List A). Then, participants will be asked to spontaneously recall the original words (List A) after a 20-min delay (i.e., long delay free recall), and finally, they will be asked to circle words from the original list (List A) in a paragraph of text containing thirty underlined words (i.e., words from the original list plus distractor words). Scores will be calculated as the total number of words recalled: 1) across the five trials (total acquisition); 2) after the interference list (recall after interference); 3) on the fifth trial minus after the interference (loss after interference); 4) at recognition (number of words correctly identified from list A); and 5) after the 20-min delay (long delay free recall – our primary RAVLT measure of interest). We will focus on changes in memory (trial completion minus baseline) over the course of the study.

#### Secondary outcomes measures

##### Comprehensive neuropsychological battery (iPad)

We will use the National Institute of Health (NIH) Toolbox Cognition Battery [40–42], a comprehensive neuropsychological battery with normative values. The cognitive battery of this toolbox includes tests that measure: *1) Executive Functions*: Executive functions is the capacity to plan, organize, and monitor the execution of behaviours that are strategically directed in a goal-oriented manner. The NIH Toolbox measures two components of executive functions: 1) inhibition and 2) set shifting. The NIH Toolbox focuses on the inhibition of automatic response tendencies that may interfere with achieving a goal. Set shifting is considered the capacity for switching among multiple aspects of a strategy or task. Inhibition will be measured with the NIH Toolbox Dimensional Change Card Sort Test. Set shifting will be measured with the NIH Toolbox Flanker Inhibitory Control and Attention Test; *2) Attention*: Attention refers to the allocation of one’s limited capacities to deal with an abundance of environmental stimulation. It is the foundation for all other types of mental processes. Attention will be measured with the NIH Toolbox Flanker Inhibitory Control and Attention Test*; 3) Episodic Memory*: Episodic memory refers to cognitive processes involved in the acquisition, storage and retrieval of new information. It involves conscious recollection of information learned within context. Episodic memory can be verbal (i.e., remembering a conversation or list of grocery items) or nonverbal (i.e., imagining a picture one saw a week ago). Episodic memory will be assessed with the NIH Toolbox Picture Sequence Memory Test. As a supplemental measure we will use the NIH Toolbox Auditory Verbal Learning Test (Rey)*; 4) Language*: Language refers to a set of mental processes that translate into symbols (words, gestures) that can be shared among individuals for purposes of communication. The NIH Toolbox focuses on two aspects of language: 1) Vocabulary knowledge, which will be measured with the NIH Toolbox Picture Vocabulary Test, and 2) Oral reading skill, which will be assessed by the NIH Toolbox Oral Reading Recognition Test; *5) Processing Speed*: Processing speed refers to either the amount of time it takes to process a set amount of information, or the amount of information that can be processed within a certain unit of time. It is a measure that reflects mental efficiency and is central for many cognitive functions and domains. Processing Speed will be measured by the NIH Toolbox Pattern Comparison Processing Speed Test; and *6) Working Memory*: Working Memory refers to a limited-capacity storage buffer that becomes overloaded when the amount of information exceeds capacity. Working Memory refers to the capacity of an individual to process information across a series of tasks, hold information in a short-term buffer, manipulate the information, and hold the products in the same short-term buffer. Working Memory will be assessed with the NIH Toolbox List Sorting Working Memory Test.

##### Executive functions

For executive functions, we will include three executive cognitive processes based on the work of Miyake and colleagues [43] and frequency of inclusion in clinical batteries [38]: 1) response inhibition, 2) set shifting; and 3) working memory. Response inhibition involves deliberately inhibiting dominant, automatic, or prepotent responses. Set shifting requires one to go back and forth between multiple tasks or mental sets [43]. Working memory involves monitoring incoming information for relevance to the task at hand and then appropriately updating the informational content by replacing old, no longer relevant information with new incoming information. We will assess: 1) response inhibition using the Stroop Colour-Word Test [44], 2) set shifting using the Trail Making Test (Parts A & B) [45]; and 3) working memory using the Digit Symbol Substitution Test (DSST; 90 s) [46].

##### Balance and mobility

The Short Physical Performance Battery (SPPB) [47] will be used to capture domains of strength, gait speed and balance, by performing standing balance, walking and sit-to-stand exercises. The SPPB is scored out of 4 points per component and has a maximum score of 12. Low scores on the SPPB reflect poor performance.

##### Cardiovascular capacity

The Six Minute Walk Test (6-MWT) [48] will be used to measure cardiovascular capacity. This test asks participants to walk as far as they can (meters) in six minutes (breaks allowed). Before and after the walk, the participants’ blood pressure will be measured. The participants will be asked to rate their walk on the Borg Rating of Perceived Exertion [33]. The score on the 6-MWT is the distance (in meters) covered during six minutes.

##### Physical activity level

To obtain information about their physical activity, the Physical Activity Scale for the Elderly (PASE) [49, 50] will be administered. This 12-item questionnaire assesses the amount of time spent per day in the previous week on leisure activity time (light, moderate and strenuous activities), household work, and time spent volunteering.

##### Magnetic resonance imaging

Prior research has demonstrated that significant changes in brain volume can be observed after 32 h of computer-based cognitive training over a span of 8 weeks among older adults with subjective memory complaints [30] – a population very similar to ours. Thus, we will include neuroimaging outcomes in our proof-of-concept RCT. Our neuroimaging outcomes will include: 1) hippocampal volume and cortical thickness as determined by structural MRI; and 2) functional connectivity as determined by resting state functional MRI and seed-based approach. If interested and eligible, a subset of participants will be asked to do one MRI scan before and one after the completion of the 8-week training. Participants will be asked to come to the UBC for 1.5 h each visit. The scanning protocol will take approximately 50 min, and a series of anatomical scans will be performed in addition to a resting-state functional MRI scan.

Acquired structural and functional neuroimaging data will be analyzed using different pipelines. The Freesurfer image analysis suite [51] will be used for structural data analysis. Freesurfer is developed at the Martinos Center for Biomedical Imaging by Laboratory for Computational Neuroimaging (http://surfer.nmr.mgh.harvard.edu/). Data processing will include skull-stripping [52], motion correction [53], Talairach transformation [54, 55], atlas registration [56] and brain parcellation [55, 57]. The data will be manually checked, and if necessary corrected. Functional connectivity analysis will be using resting-state functional MRI (rsfMRI) data to investigate the effect of CCT (alone and preceding a 15-min walk) on functional connectivity. Resting-state fMRI data will be preprocessed using FSL (FMRIB’s Software Library). Data processing will include skull-stripping using Brain Extraction Tool (BET), motion correction using MCFLIRT, and spatial smoothing. Data will be manually checked, and if necessary corrected. Model-free independent component analysis (ICA) will be performed using FSL-MELODIC to examine whole-brain connectivity patterns, and with selecting independent resting-state components, we will look at between group differences. Seed-based functional connectivity analysis (SBA) will be performed to look at the correlations between regions of interest within and between networks. Connectivity maps will be created to show connections with the seed region (i.e., region of interest).

### Participant timeline

**Fig. 2:**
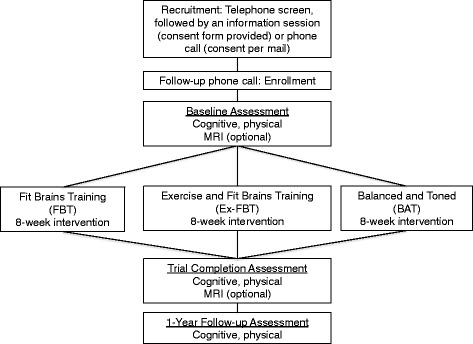
Participant timeline

Eligible participants will attend a 1-hour information session at either the UBC or at VGH. During this one-hour information session, the study coordinator will give a short presentation that provides the potential participants with important details of the study. In addition, the potential participants will receive a copy of the consent form during this visit. Once written consent is obtained, a research assistant will schedule a baseline assessment. After completion of their baseline assessment, participants will be randomized into one of 3 training groups (i.e., FBT, Ex-FBT, or BAT). Following the 8-week intervention, participants will attend the final assessment session(s). For a complete timeline, see Fig. 2.

### Sample size and randomization

The required sample size for this study is calculated based on changes in the RAVLT (retention score). Specifically, we predict a mean change of 0.31 for the FBT group, a mean change of 0.40 for the Ex-FBT group, and a mean change of −0.31 for the BAT group. We made these estimates based on the work of Diamond and colleagues [58]. With a pooled standard deviation of 1.1, and alpha of 0.05, we will need 36 participants for a power of 0.80. To accommodate for a 10% drop-out rate, our total sample size comes to 120 participants (i.e., 40 FBT, 40 Ex-FBT, and 40 BAT).

Participants will be randomly allocated (1:1:1) to FBT, Ex-FBT, or BAT. The randomization sequence will be generated by an independent member of the team using computer software (www.randomization.com). Blocked randomization will be used, with a block size of 12. The group allocation will be concealed for the study coordinator. After enrolment, performed by a research assistant, and completion of the baseline assessment, the study coordinator will send a list of participant identification numbers to the independent member responsible for the randomization. This independent member will provide the study coordinator with the group assignment for the enrolled participants. After completion of baseline assessment at VGH, the participants will be informed of their group assignment. Outcome assessors will be blinded after treatment allocation.

### Adverse events monitoring

Adverse events will be monitored using adverse event forms. All adverse events will be discussed with the principal investigator and the study team to see whether any adaptations to the protocol or program should be made as a result of the adverse event and to insure safety for all participants.

### Data management

Data will be entered ongoing over the study period. Data will be securely stored in a locked cabinet and in a secured online database. Random data checks will be performed to promote data quality.

### Statistical analysis

#### Effects of CCT

The primary and secondary outcomes will be analyzed using an identical analytic model, which will follow the intention-to-treat principle, such that all randomized participants will be included to estimate treatment effects irrespective of deviations from treatment protocol (e.g., loss to follow-up, non-compliance). This will be done using linear mixed models using maximum likelihood estimation. The model will include random intercepts, and fixed effects of time (baseline, trial completion), intervention assignment (FBT, Ex-FBT, BAT), and their interaction. Baseline MoCA score and age will also be included as fixed effect covariates. Treatment effects will be indicated by a statistically significant treatment by time interaction. Two planned simple contrasts will be performed to assess differences in changes in the primary and secondary outcomes between: 1) the FBT group and the BAT group; and 2) the ex-FBT group and the BAT group. A secondary planned contrast will determine whether FBT and ex-FBT will differ in changes in the primary and secondary outcomes over time. To explore maintenance of treatment effects, we will perform repeated measures with linear mixed models using maximum likelihood estimation. The models will include random intercepts, and fixed effects of time (baseline, trial completion, 1-year follow up), intervention assignment, and their interaction. Baseline MoCA score and age will also be included as fixed effect covariates.

Follow-up sensitivity analyses will restrict the study sample to individuals with valid data at all three time points (baseline, trial completion, and 1-year follow-up). The same linear mixed models describe above will be employed to determine whether inferences will be similar for the intention-to-treat and complete-case study samples.

#### Baseline cognitive status as a moderator

To determine whether treatment effects are similar for individuals identified as having MCI, we will add MCI status as an additional fixed effect in the linear mixed models described above. Moderation will be indicated by a statistically significant MCI status by treatment by time interaction. In the presence of moderation, the planned contrasts described above will be re-computed after stratifying by MCI status. This will identify how MCI status moderated the effects of treatment on the outcome of interest.

## Discussion

Currently there are a limited number of high quality studies investigating the efficacy of CCT programs; therefore findings from this randomized controlled trial will contribute to the existing research. In addition, a gap currently exists in literature investigating the effect of these programs in an older adult population with MCI. If this research demonstrates benefits of an 8-week CCT intervention, both short-term (i.e., trial completion) and long-term (1-year follow-up), CCT might serve as an easy accessible strategy to combat cognitive decline in healthy older adults and as a potential effective way to alter the trajectory of cognitive decline in older adults with MCI.

### Neural mechanisms

Evidence regarding the underlying neural mechanisms of CCT in both healthy older adults and older adults with MCI is limited. If the current study provides evidence of changes in neural structure or neural activity (e.g., functional connectivity), it would be a considerable contribution to research in this field.

## Abbreviations

6-MWT: 6-Minute Walk Test
BAT: Balanced and Toned
BET: Brain Extraction Tool
CCT: Computerized Cognitive Training
CONSORT: Consolidated Standard of Reporting Trials
Ex-FBT: Exercise plus Fit Brains Training
FBT: Fit Brains Training
FCI: Functional Comorbidity Index
FSL: FMRIB’s Software Library
IADL: Instrumental Activities of Daily Living
ICA: Independent Component Analysis
MCI: Mild Cognitive Impairment
MMSE: Mini-Mental State Examination
MoCA: Montreal Cognitive Assessment
MRI: Magnetic Resonance Imaging
NIH: National Institute of Health
PASE: Physical Activity Scale for the Elderly
RAVLT: Rey Auditory Verbal Learning Test
RCT: Randomized Controlled Trial
rsfMRI: resting-state functional Magnetic Resonance Imaging
SBA: Seed Based Analysis
SPIRIT: Standard Protocol Items Recommendations for Interventional Trials
SPPB: Short Physical Performance Battery
UBC: University of British Columbia
VGH: Vancouver General Hospital
